# Effects of small airtime rewards linked to unsolicited text messages on uptake of a tuberculosis self-screening app in South Africa: a randomised trial

**DOI:** 10.1136/bmjopen-2024-097650

**Published:** 2026-01-27

**Authors:** Kate Rich, Ronelle Burger, Andrew Boulle, Deanne Goldberg, Noxolo Gqada, Juan-Paul Hynek, Adam Loff, Pren Naidoo, Desi Nair, Arne von Delft, Matthias Rieger

**Affiliations:** 1 Department of Economics, Stellenbosch University, Stellenbosch, South Africa; 2 School of Economics and Finance, University of the Witwatersrand, Johannesburg, South Africa; 3 School of Public Health and Family Medicine, University of Cape Town, Rondebosch, South Africa; 4 Department of Health and Wellness, Western Cape Provincial Government, Cape Town, South Africa; 5 Clinton Health Access Initiative, Johannesburg, South Africa; 6 Reach Digital Health, Cape Town, South Africa; 7 Public Health Management Consultant, Cape Town, South Africa; 8 Erasmus School of Health Policy & Management, Erasmus University Rotterdam, Rotterdam, Netherlands; 9 International Institute of Social Studies, Erasmus University Rotterdam, The Hague, Netherlands

**Keywords:** Tuberculosis, Digital Technology, Self-Help Devices, Mass Screening

## Abstract

**Objectives:**

The objective of this study was to test whether an airtime reward increased tuberculosis (TB) Check screening uptake. This served as a feasibility study for the planned Phase 2, which aimed to test behavioural messaging to boost take-up of TB testing among users who were advised to get tested by TB Check.

**Design:**

The study was a randomised controlled trial with a parallel design.

**Setting:**

This study assessed mHealth support to boost TB testing in high-burden Cape Town clinics.

**Intervention:**

Patients aged 18 or above with a valid mobile phone number that had been added within the last 5 years were invited by the Western Cape Department of Health and Wellness through unsolicited text messages to screen for TB using TB Check.

**Participants:**

Patients in the intervention group (n=1250) were additionally offered R15 airtime for completing the screening and participating in the research study. Patients were allocated to the intervention or control group through parallel randomisation with equal group size.

**Primary outcome measures:**

The primary outcome was the number of TB Check screenings completed within 1 week of the SMS invitation being sent.

**Results:**

Messages were successfully delivered to 616 patients in the control group and 633 patients in the intervention group. Uptake of the invitation by the intended recipients was very low. Eight users in the control group and 20 users in the intervention group initiated a self-screening (1.3% vs 3.2% of delivered messages; 95% CI of difference (0.2 to 3.5)), but only three users in the control group and seven users in the intervention group successfully completed a self-screening (0.49% vs 1.11% of delivered messages; 95% CI of difference (−0.4 to 1.6)). Low delivery of text message invitations (50.0%) and low completion of users who started the screening (35.7%) posed additional challenges. No adverse events were recorded.

**Conclusions:**

The addition of a small airtime participation reward to unsolicited text message invitations did not appear to be an effective tool to reach targeted individuals in this context. The results of Phase 1 reported here suggested that Phase 2 would not be feasible, so we did not proceed with the planned Phase 2. However, uptake of incentivised self-screening was unexpectedly high among users who were not originally invited (presumably known contacts of the original invitees). Within 5 days of the invitations being sent, 1962 unique self-screenings had taken place using the incentive code; only 7 of these users were originally invited. The lessons learnt from this study can help to inform future efforts to promote TB self-screening, mHealth initiatives and attempts to engage with patients via text message.

**Trial registration:**

The study was pre-registered with the South African National Clinical Trials Registry (Phase 1 trial no DOH-27-112023-9045, Phase 2 trial no DOH-27-112023-4944) and the Pan African Clinical Trials Registry (Phase 1 trial no PACTR202311529334858).

**STRENGTHS AND LIMITATIONS OF THIS STUDY**

- The randomised design provides high-quality evidence.
- The study documents evidence on real-world challenges involved in using mHealth applications to promote self-screening for infectious disease, as well as in using text messages to contact patients in a low- and middle-income country public healthcare system.
- The airtime reward (R15, ~US$0.80 or 60% of the minimum hourly wage in South Africa at the time) may not have been sufficiently large to encourage uptake of the screening tool.

## Introduction

Tuberculosis (TB) was the leading cause of death in South Africa in 2018.^1^ South Africa is among the 30 countries with the highest burden of TB,^2^ accounting for 3.6% of the global TB burden.^3^ According to South Africa’s first national TB prevalence survey conducted in 2018, South Africa had roughly 150 000 ‘missing’ TB cases—of the estimated 390 000 people with TB in 2018, only about 240 000 had been diagnosed.^4^ Timely case detection is crucial to avoid treatment delays, which are associated with higher morbidity and mortality.^5^

TB Check is a self-screening service designed to boost case detection at scale. The WhatsApp- and USSD-based conversational platform is supported via the National Department of Health’s contact line. After answering a series of screening questions related to their symptoms and TB risk, users classified as high risk are advised to visit their local clinic for a free TB test. Users can also access information about TB symptoms, testing and treatment through the platform.

The service was launched by the National Department of Health in October 2020, but take-up was fairly low relative to the population of ‘missing’ TB cases. Over the 2-year period from the launch of the platform until 2 October 2022 (the day before additional promotional campaigns began, evaluated in a separate paper), 71 787 completed self-screenings from a total of 55 461 unique users were recorded, of which 60% of total screens were classified as high-risk and/or symptomatic (this period covered several waves of the COVID-19 pandemic). ‘Users’ refers to individual cellphone numbers throughout. Some people may have access to more than one cellphone; alternatively, more than one person may share a single cellphone.

Some studies—including from low- and middle-income countries (LMICs)—suggest that fairly small financial incentives can increase HIV testing^6 7^ and HIV+case detection,^7^ as well as increase screening for cancer.^8^ Research participation rewards have also been shown to increase participation in randomised controlled trials.^9^ However, there is limited evidence on the effectiveness of small financial incentives offered through unsolicited text messages (SMSes) for promoting TB self-screening.

Text messages have been effective in promoting health behaviours in some contexts. Text message reminders have been shown to increase influenza vaccination rates in the USA.^10^ In the South African context, text message reminders led to more timely collection of TB test results,^11^ but the effectiveness of text messages sent unsolicited by public healthcare providers may differ from that of messages sent to participants who have already agreed to enrol in a study. We are also not aware of any studies on the effectiveness of text messages coupled with small financial incentives to promote TB screening. Accordingly, the aim of this study was to evaluate the effect of offering a small airtime reward linked to unsolicited text messages on take-up of TB self-screening using the TB Check platform. This study contributes to the limited evidence on the real-world effectiveness and challenges of unsolicited text message contact strategies for engaging with populations at risk for health promotion and prevention in the public health system in LMICs.

## Methods

### Study design and setting

The randomised controlled trial was conducted in partnership with the Western Cape provincial Department of Health and Wellness (WCDHW), with unsolicited invitations sent by the public healthcare system to known patients for whom it was considered by the healthcare provider that TB self-screening would be in the interests of their personal health.

The study was planned in two phases. Phase 1 tested whether an airtime reward linked to unsolicited text messages increased TB Check screening take-up relative to the control via a simple comparison of take-up rates. Phase 1 served as a feasibility study for Phase 2, which aimed to test small behavioural devices to boost take-up of TB testing among users who were advised to get tested by TB Check. Phase 2 was planned to proceed if the results of Phase 1 suggested that take-up rates were sufficient to power Phase 2 without sending a prohibitively large number of text message invitations.

### Intervention: Phase 1

Patients in the control group received the message:

“The WC Dept of Health encourages citizens to screen themselves for TB by dialling *134*832*57# (free of charge) or WhatsApp 'Go' to 0600123456 bit.ly/46583jW”

Patients in the intervention group received the message:

“The WC Dept of Health encourages citizens to screen themselves for TB by dialling *134*832*52# (free of charge) or WhatsApp 'Wc’ to 0600123456. When you complete the screening, you will receive R15 in airtime from our research partner Stellenbosch University for participating in the linked research study bit.ly/47impyy”

The R15 airtime was framed as a research participation reward paid by the WCDHW’s research partners, Stellenbosch University. The airtime reward was sent to users who completed a screening an hour after completing the screening.

Both messages included a shortened link to the WhatsApp platform, which would take the user directly to the WhatsApp account with the correct keyword without them having to type in the phone number or add it as a contact. The study arms had different keywords used to access the platform in the case of WhatsApp, and different USSD codes in the case of USSD. The keywords and numbers were the same length in both cases.

The text message invitations were sent on 9 November 2023. The study was closed on 14 November, after which users could still complete a screening but were notified that the study had been closed and they would no longer receive airtime. The text message invitations were translated, reviewed by native speakers and then sent in English, Afrikaans or isiXhosa, the main languages used in the Western Cape, depending on the user’s recorded language preference. If no preferred language was recorded, the message was sent in English. Each mobile phone number was restricted to participate in the study once. If a second screening attempt was received from a mobile phone number, the user was informed that they could only participate in the study once, but that they could proceed to use TB Check to screen themselves for TB. Users were asked for their age group, and users under 18 were informed that they could not participate in the study but could still continue to screen themselves and were re-directed to the routine version of TB Check.

### Intervention: Phase 2

Phase 2 aimed to test the impact of behavioural messaging and small airtime payments on our main outcomes: TB test take-up and clinic visits after TB Check self-screening. Users’ risk for TB would be assessed with a list of screening questions and those assessed to be at risk for having TB would have been recommended to go for a TB test. After being advised to go for a TB test, the intervention group would have been offered an additional R15 if they committed to get tested for TB, while the control group would have received the standard message requesting them to go for a TB test. The intervention group would also have received a message prompting them to plan when they would go for a test (see online supplemental materials).

### Participants

A total of 2500 unique patients were invited by unsolicited text message by WCDHW to self-screen using TB Check. Numbers were selected randomly from a list of patients in the WCDHW patient database, based on the determination that it would be in the interests of their own health to screen for TB. Randomisation was at the individual level. The list of patients from which the random sample was drawn was limited to patients with a mobile phone number that had been added within the last 5 years (since 2018), with a syntactically valid mobile phone number (valid cellular prefix and length), and who were above the age of 18.

Power calculations were informed by an operational pilot prior to the experiment. On March 15 2023 and before the RCT, 1000 test invitations (control message without incentives) were sent out. This was to test delivery success and screening uptake. No incentive was tested and these data only informed the power calculations. Out of these 1000 test invites, 46% were successfully delivered and 2% (out of 463 deliveries) led to the initiation of a screening, respectively. For the RCT experiment, we assumed a minimum detectable impact of 4 percentage points (uptake rates among delivered messages: control 2% vs intervention 6%) induced by incentives as this was deemed the minimal, policy-relevant effect size by our team of researchers and stakeholders. We used Stata’s *power* command with power of 80% and a significance level of 5%. The power calculations yielded a sample size of 376 per group. This group size was divided by the SMS delivery success rate of 46% and multiplied by the initially planned three intervention groups to get a sample size of 2452. This was rounded up to a final sample size of 2500. We later decided to simplify the experiment to include only two experimental groups (a control group plus one intervention group), but kept the initial sample size, so each experimental group had 1250.

From these 2500 patients, 1250 numbers were individually randomised to receive the control message and 1250 to receive the intervention message. Computer-based simple, parallel randomisation with equal group size was used to allocate numbers to the intervention and control groups, and was carried out by the WCDHW.

### Outcomes

The primary outcome was the number of TB Check screenings completed within 1 week of the SMS invitation being sent, and the secondary outcome was the number of TB Check screenings initiated within 1 week of the SMS invitation being sent. These outcomes were available from the TB Check usage data collected by the developer, Reach Digital Health.

### Statistical analysis

We tested for differences in the proportion of messages delivered across the intervention and control group using the χ^2^ test. We tested for differences in the number of users initiating and completing a screening across the two groups (as a percentage of the total messages sent and as a proportion of the messages delivered) using Fisher’s exact test, as the numbers in question were very small. As additional exploratory analyses, we examined what point in the screening users who began a screening but did not complete it stopped at and examined the number of users who did not receive the SMS invitation directly from the WCDHW but completed a screening using the intervention group code. All statistical analyses were carried out using Stata version 18.

### Regulatory approvals

To preserve patient privacy, data was extracted, de-identified and analysed by the Western Cape Provincial Health Data Centre team. Only aggregated results were shared with the research partners. The study was pre-registered with the South African National Clinical Trials Registry (Phase 1 trial no DOH-27-112023-9045, Phase 2 trial no DOH-27-112023-4944) and the Pan African Clinical Trials Registry (Phase 1 trial no. PACTR202311529334858) in accordance with WHO and ICMJE standards.

### Patient and public involvement

Neither patients nor the public were involved in the design or conduct of the study.

## Results

**Figure 1:**
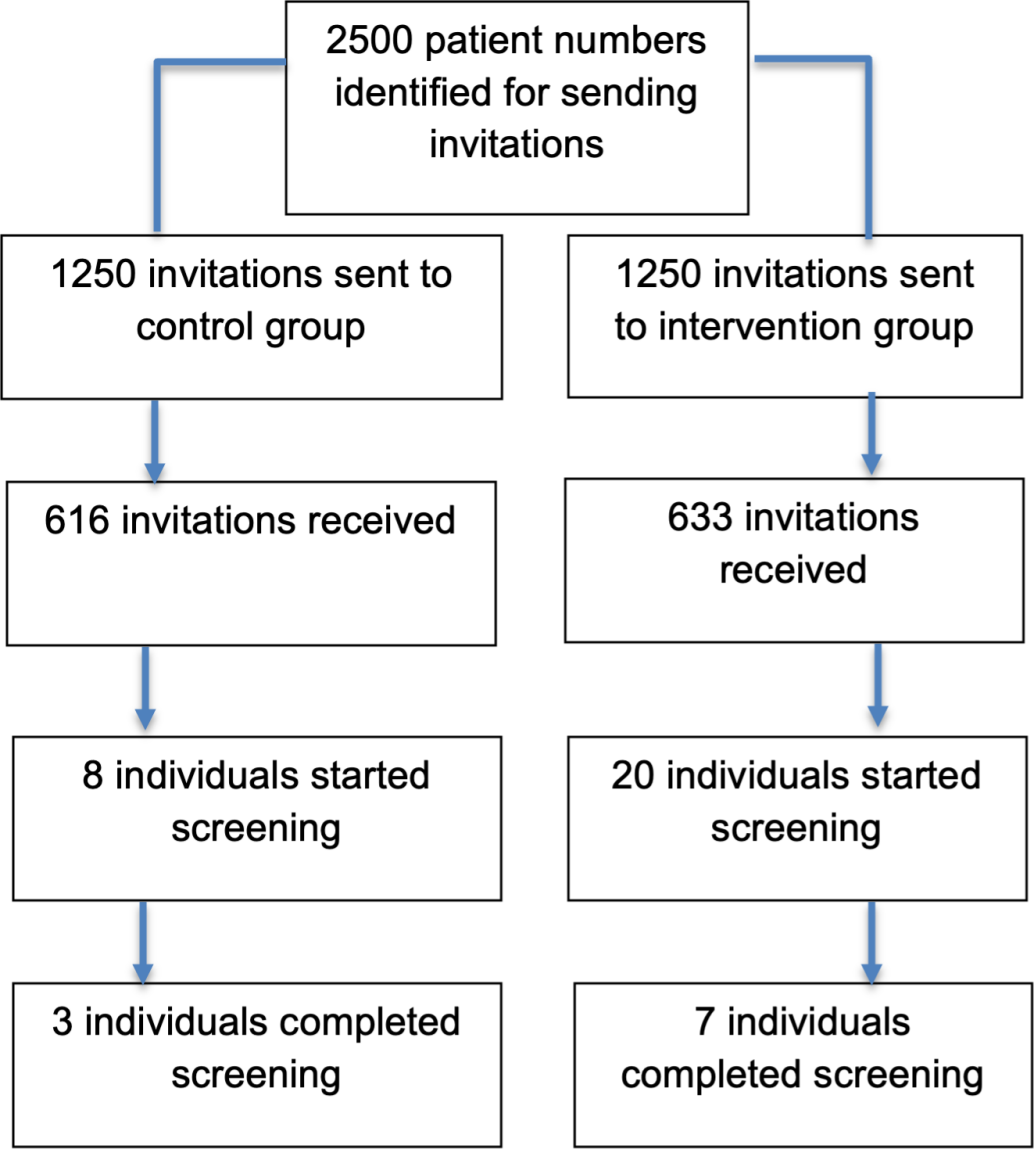
Data flow diagram.

Table 1 and figure 1 show that 616 (49.3%) of the 1250 control group and 633 (50.6%) of the 1250 intervention group text message invitations were successfully delivered.

**Table 1:** Messages delivered, screenings begun and screenings completed in intervention and control groups

	Control N (%)	Intervention N (%)	RR (95% CI)	Difference in proportions (95% CI)	P value of difference in proportions
Invitations sent	1250	1250			
Device acknowledged delivery (callback)	616 (49.3%)	633 (50.6%)	1.03 (0.95 to 1.11)	1.4 percentage points (−2.6 to 5.3)	0.497
Unique users beginning a screening	8 (1.3% of delivered messages)	20 (3.2% of delivered messages)	2.50 (1.09 to 5.73)	1.9 percentage points (0.2 to 3.5)	**0.035**
Unique users completing a screening	3 (0.5% of delivered messages)	7 (1.1% of delivered messages)	2.33 (0.60 to 9.09)	0.6 percentage points (−0.4 to 1.6)	0.342
Percentage of users beginning a screening who completed a screening	37.5%	35%		−2.5 percentage points (−42.0 to 37.0)	1.000

All p values are two-sided. To test the difference in the proportion of messages delivered, we used the χ2 test. The rest of the p values are based on Fisher’s exact test, as the numbers in question were very small. P values in bold font indicate statistical significance at the 5% level.

20 users in the intervention group initiated a self-screening (3.2% of delivered messages), compared with eight in the control group (1.3% of delivered messages; 95% CI of difference (0.2 to 3.5); Fisher’s exact p value=0.035). However, only seven users in the intervention group and three users in the control group successfully completed a screening. This represents 1.1% of delivered messages in the intervention group compared with 0.5% of delivered messages in the control group (95% CI of difference (−0.4 to 1.6); Fisher’s exact p value=0.342) and 0.6% versus 0.2% of sent messages (95% CI of difference (−0.2 to 0.8); Fisher’s exact p value=0.343).

In the control group, 3 of 8 (37.5%) unique users who started a screening completed it. In the intervention group, 7 of 20 (35%) unique users who started a screening completed it. In the control group, 37.5% of users who began a screening stopped during the preliminary questions before getting to the symptom questions, while 25% stopped during the symptom questions. In the intervention group, half of the 20 users who began a screening stopped before getting to the symptom questions, 10% stopped during the symptom questions and 5% timed out. However, there was no clear pattern in where users stopped the screening during the preliminary questions. Only one user across the two groups stopped at the research consent phase.

**Figure 2:**
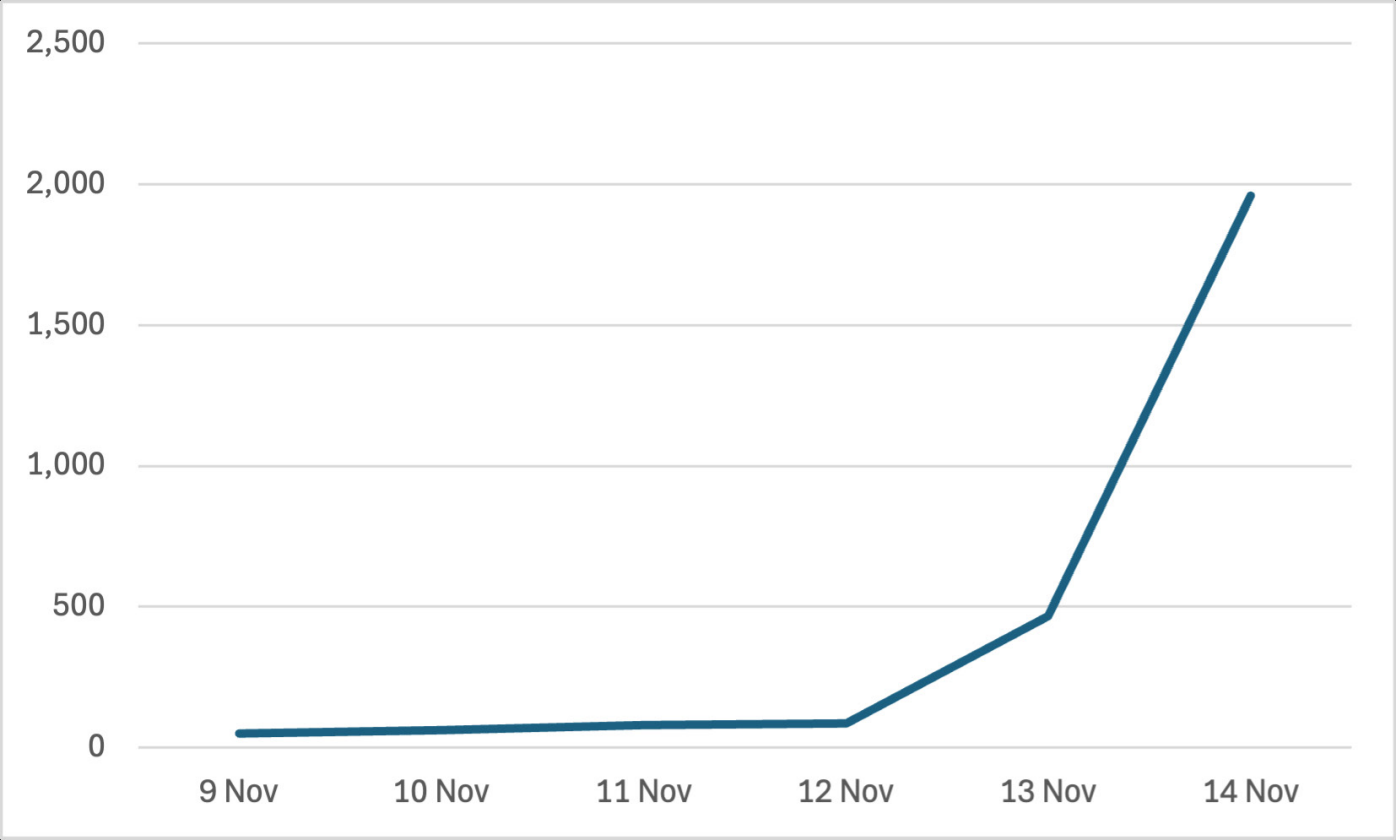
Cumulative number of TB Check screenings per day by unique users using the intervention group code in the days following text message invitation, 9–14 November 2023.

Figure 2 shows the number of completed TB Check self-screenings per day by unique users who accessed the platform using the intervention group keyword or USSD code in the days following the sending of the text message invitation. Incentivised self-screenings rose from 51 on 9 November, the day the invitations were sent, to 1962 cumulative self-screenings by distinct users by 14 November. Since only seven invited users in the intervention group completed a self-screening, these additional 1955 ‘uninvited’ incentivised self-screenings probably resulted from the forwarding of text message invites or the sharing of USSD information, most likely in response to receiving the airtime reward. Some users may also have completed a screening again on another cellphone or number. Three screens were completed using the control group code overall, suggesting that no further forwarding or sharing took place in the control group. The completion of incentivised self-screenings by users who were not initially invited was an unintended effect, but as users still had to consent to use the application, there was no harm involved.

After reviewing results from Phase 1, take-up by invited participants was deemed too low to power Phase 2 without sending a prohibitively large number of text message invitations, so we did not proceed with the planned Phase 2. Self-screenings by participants who were not originally invited could not be used for Phase 2 because they could not be linked to clinical data on TB testing.

## Discussion

Even with the addition of a small airtime participation reward, unsolicited text messages do not appear to be a cost-effective tool to promote targeted TB self-screening in South Africa. In absolute terms, take-up was very low among the intended recipients given the number of text message invitations sent. However, uptake of incentivised self-screening was much greater in response to the apparent sharing of text message invites beyond people who were initially invited, presumably among trusted contact networks.

The low uptake of TB Check by invited participants in response to the unsolicited text message invites may be due to the low salience of text messages, which warrants further investigation and alternative contact strategies. While our messages were unsolicited, invitations were sent to people who already had a relationship with the WCDHW. Still, given the number of marketing and spam text messages commonly received, people may be inclined to ignore or pay little attention to text messages. A study in Indonesia found that personalised text messages (using the recipient’s name) improved health screening uptake,^12^ and a meta-analysis found that personalised text message interventions were generally more effective for health promotion than non-personalised interventions.^13^ Our text message invitations may have been less salient because they were not personalised. The text messages were sent by a Department of Health number that would not have been saved as a known and trusted contact by the recipients. Recipients were also not informed that they might be getting a text message from the Department and were not offered the opportunity to opt out. Recipients may thus not have trusted that the text message invitations were legitimate or even have read them in the first place. We do not know how many participants read the messages and therefore knew about the incentive. Speculatively, the high number of self-screenings by users who were not originally invited but presumably received the invitation from a trusted contact, in comparison with the very low uptake among invited users who received the invite from an unknown sender, suggests that uptake may be higher when an invite comes from a trusted source.

The low delivery success of the text message invitations suggests that the quality of contact details in patient databases poses a possible challenge to communicating with patients in the public healthcare system via text message in this context. Even though the sample was limited to patients whose contact details were added in the previous 5 years and were syntactically valid, half of the messages sent could not be delivered, possibly because the mobile phone numbers had expired or because the numbers were incorrect. Other South African studies using text messages have also experienced fairly high non-delivery, though none were as high as in this study. In a study of a text message programme to improve HIV antiretroviral therapy adherence in another province of South Africa (Kwa-Zulu Natal), 72.7% of treated patients had at least one text message successfully delivered.^14^ In that study, phone numbers had been added at clinic enrolment, an average of 3.3 years before the text message programme began. A study testing text message reminders to return to collect TB test results, also in the Western Cape public healthcare system, had a non-delivery of 15%.^11^ In that study, patients’ mobile phone numbers had been collected less than a week prior to sending text message reminders, so it would be expected that delivery success would be considerably higher than in this study which included numbers from 5 years earlier. The low delivery success for recently collected phone numbers shows that outdated phone numbers are not the only constraint and that accurate recording and verification of phone numbers are vital.

The low completion by users who started a screening (35.7% across intervention and control groups) suggests that privacy concerns related to the stigma around TB and network instability may be barriers to usage of mobile self-screening platforms in LMICs. TB stigma—in conjunction with concerns around confidentiality—has been identified as a barrier to accessing diagnostic services in the South African context.^15^ However, as discussed above, 46% of users who began a screening stopped at one of the preliminary questions—asking users to accept the terms and conditions (with an option to request more information), accept the platform privacy policy, consent to participate in the research and asking what gender they identify as. Given the stigma surrounding TB among many South Africans, it is plausible that observed hesitance may be due to privacy concerns. Privacy concerns may be exacerbated in a context where people commonly share a phone with other family members. Alternatively, it may also be that users were impatient and overwhelmed with the number of questions (the USSD version included 17 prompts; the usual number is around 4). When it comes to communicable diseases, it is vital to minimise errors of exclusion, so it is important to remove any impediments to user onboarding. Network instability in areas of poor mobile phone reception (or due to the planned power outages common in South Africa) may also have led to screenings being interrupted, particularly on the USSD platform where most screenings took place. Although users could resume a screening where they left off without having to start from the beginning, some users may not have attempted to resume their self-screening after being interrupted.

Take-up was particularly low on the WhatsApp platform. Only one unique invited user began a screening on WhatsApp, and there were no successful completions on WhatsApp. It is possible that recipients had additional privacy concerns about sharing their health information on WhatsApp. Another possibility is that users were not able to screen on the WhatsApp platform due to a lack of access to cellular data or WiFi. Although users were offered airtime rewards for completing a screening, users who did not have any airtime or cellular data to begin with would not have been able to initiate a screening on WhatsApp.

The sharing of text message invites in response to the airtime reward reached a large number of users who were not initially invited. This could potentially prove useful in some contexts, such as for incentivised contact tracing. Unfortunately, we could not observe how many of the originally invited recipients shared the invite message with their contact networks, or how many people ultimately received the invite, so we could not calculate uptake among users who were not initially invited or explore this sharing further. 55% of total incentivised self-screenings in this study (including users who were not initially invited) were high-risk or symptomatic; these users were recommended to go for a TB test. However, users who were not invited directly by the WCDHW could not be linked to clinical TB testing data, as their numbers were not necessarily present in the WCDHW patient database, so it is unclear whether these incentivised self-screenings resulted in more TB tests.

This study was subject to a number of limitations. First, R15 (roughly US$0.80 on 9 November 2023) may not be enough reward for a large share of our sample. In South Africa, the minimum hourly wage in November 2023 was R25. Second, we cannot observe whether the linkage to research may have discouraged take-up. Third, although we aimed to make the invitation messages clear and easy to understand, limited literacy may have posed a barrier to uptake of the screening tool or contributed to heterogeneity in the incentive effect. Fourth, we report simple differences between the randomised treatment and control groups but were unable to assess or adjust for any baseline imbalances. Finally, the study was limited to adults aged 18 and older.

## Conclusion

Even with a small airtime reward, targeted take-up of TB self-screening in response to an unsolicited text message invitation was extremely low. This study highlights challenges to mobile health (mHealth) self-screenings in a public healthcare system, including low delivery success of text message invitations, low targeted take-up and low screening completion. The opportunities presented by the unexpectedly widespread uptake of incentivised self-screening among trusted internal networks should be explored further. We hope that the lessons learnt from this study can help to inform future efforts to promote self-screening and prevention via innovative mHealth initiatives.

## Supplementary material

10.1136/bmjopen-2024-097650 online supplemental file 1

## Data Availability

No data are available.
