# Supplementary material for: Effects of small airtime rewards linked to unsolicited text messages on uptake of a tuberculosis self-screening app in South Africa: a randomised trial
Source: BMJ Open. 2026 Jan 27;16(1):e097650. doi: 10.1136/bmjopen-2024-097650 (PMC12853474; doi:10.1136/bmjopen-2024-097650)
Supplement: online supplemental file 1 [file bmjopen-16-1-s001.docx]

**Online supplementary material**

*Phase 2 behavioural messages*

**
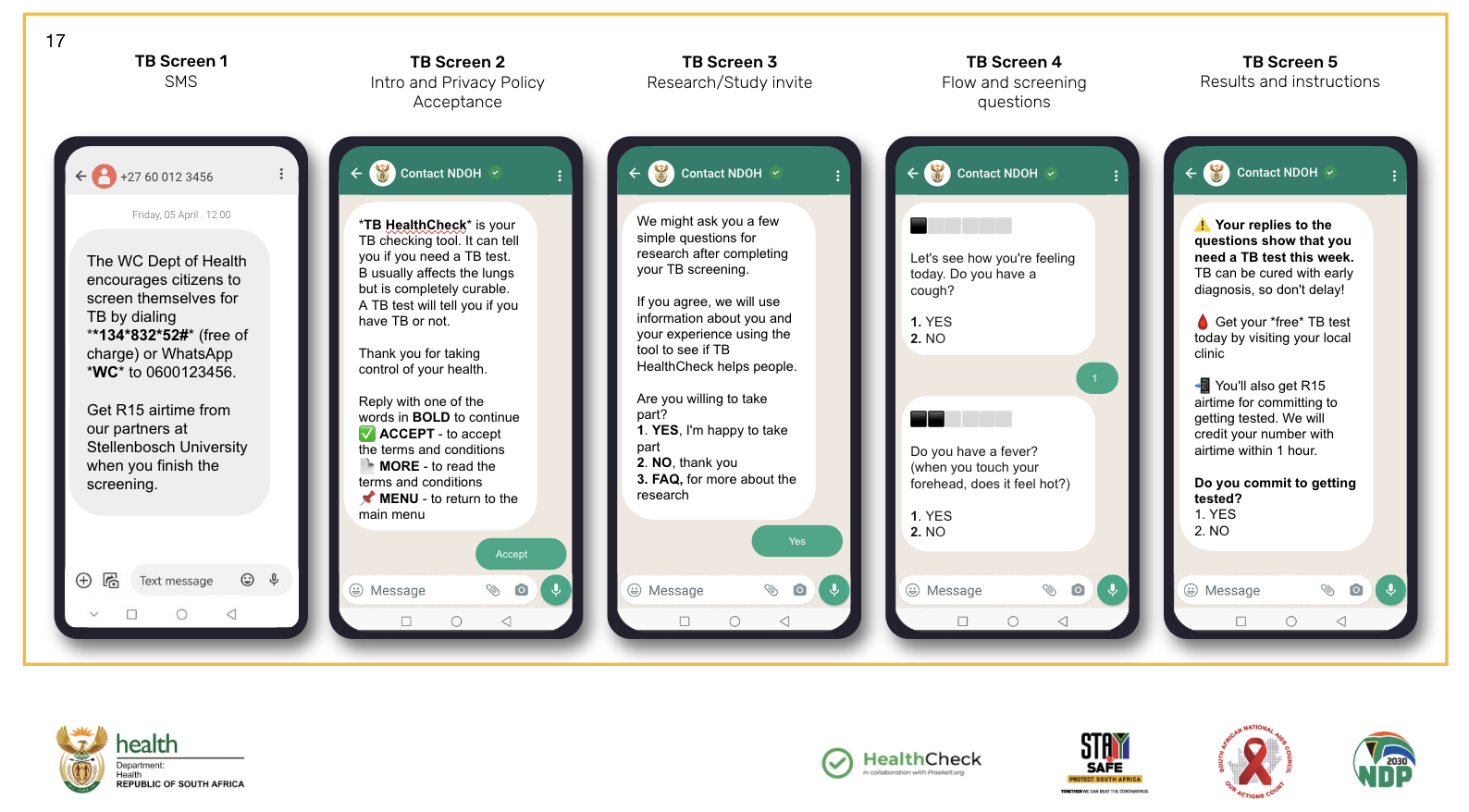
**

The control message, as on the existing platform, was:

“⚠️ Your replies to the questions show that you need a TB test this week.

🩸 Visit your local clinic for a free TB test.”

The intervention message was:

“⚠️ Your replies to the questions show that you need a TB test this week.

With early diagnosis, TB can be cured. Don’t delay, test today!

🩸 Visit your local clinic for a free TB test.

📲 You will get R15 airtime if you commit to get tested. Airtime will be sent to you within 1 hour.

Do you commit to getting tested?

1. YES

2. NO

Do you know which facility you will visit? If not you can look up the facilities in your community using this link: www.westerncape.gov.za/static/health-facilities/

Get there early! Clinics are open for TB testing Monday to Friday mornings.

Many people find it helpful to make a plan for when to go and get tested. Plan when you will go and get tested by selecting a day below:

1. MONDAY

2. TUESDAY

3. WEDNESDAY

4. THURSDAY

5. FRIDAY”
