# Supplementary material for: Effects of small airtime rewards linked to unsolicited text messages on uptake of a tuberculosis self-screening app in South Africa: a randomised trial
Source: BMJ Open. 2026 Jan 27;16(1):e097650. doi: 10.1136/bmjopen-2024-097650 (PMC12853474; doi:10.1136/bmjopen-2024-097650)
Supplement: Supplementary file 2 [file bmjopen-2024-097650.reviewer_comments.pdf]

PEER REVIEW HISTORY

BMJ Open publishes all reviews undertaken for accepted manuscripts. Reviewers are asked to complete a checklist review form and are provided with free text boxes to elaborate on their assessment. These free text comments are reproduced below.

ARTICLE DETAILS

Title (Provisional)

Effects of small airtime rewards linked to unsolicited text messages on uptake of a tuberculosis self-screening app in South Africa: a randomised trial

Authors

Rich, Kate; Burger, Ronelle; Boulle, Andrew; Goldberg, Deanne; Gqada, Noxolo; Hynek, Juan-Paul; Loff, Adam; Naidoo, P.; Nair, Desi; von Delft, Arne; Rieger, Matthias

---

VERSION 1 - REVIEW

**Reviewer**	**1**
**Name**	**Tumuhimbise, Wilson**
**Affiliation**	**Mbarara University of Science and Technology**
**Date**	**08-May-2025**
**COI**	**None**

Page 9, lines 31-34, was that 14th November same year 2023, what was the reason for that short time

Frame, was this a design issue and could this have had a significant effect on the study since participants were told that they wouldn't receive the airtime?

Page 13, line 3 and 5, define the term unique users

The fifth bullet under the strengths and limitations on

Table 1, Is true that this entire study only 27 people in the intervention arm and 11 people in the Control arm began and completed the screening using the mobile phone out of the 1249 devices that acknowledged? Was the unsolicited by design or coincidental ? Given that people will always participate in studies they understand. could this have contributed to this small number otherwise the effect of a good intervention like this may be deemed not effective because of the researchers' bias. When we consider the short time frame given and the probably

---

**Reviewer**	**2**

**Name**	**Afşin, Emine**
**Affiliation**	**Bolu Abant İzzet Baysal University, chest diseases**
**Date**	**29-May-2025**
**COI**	**None**

Although the aim was to detect TB early, the number of patients who completed the screening was very low and the limitations mentioned indicate that this study needs to be further developed.

**Reviewer**	**3**
**Name**	**Moinuddin, Mohammed**
**Affiliation**	**University of Lancashire, School of Medicine and Dentistry**
**Date**	**04-Sep-2025**
**COI**	**None**

Statistical reviewer's comment

Results from a randomized controlled trial to evaluate whether airtime rewards through unsolicited text messages increase the uptake of the TB Check self-screening platform. This is an interesting research topic; however, there are several caveats regarding the methodology and statistical analysis that require further attention. My specific observations are below.

Comment 1: Sample size calculation and sampling - The authors mentioned "a power of 80%, a significance level of 5%, assuming a screening completion rate of 2% in the control group and 6% in the intervention group." Please clarify how you chose the 2% and 6% baseline rates. Please provide the reference for this statement. Please briefly explain.

The authors again mentioned in the footnote that the sample size was calculated for three groups with a success rate of 46%, which gives a total of 2452. However, the original description states that it was calculated based on a 2% success rate, with the number showing the same. I wonder how these two descriptions make sense. Please clarify.

Non-response in the form of non-delivery of text messages in this study was a major issue. Unfortunately, the authors did not consider this in their sample size calculation. Please justify.

It is surprising how the randomization allocated the same number of patients in the two groups. This usually does not happen in simple randomization unless some mechanisms, such as block randomization, are adopted. In addition, please describe the participant selection procedure in detail.

Comment 2: I wonder whether all the participants had sufficient language capability to understand the text messages. Please describe and compare the language by use and literacy rates by group in the study region. This may have been a strong confounder in this study.

Comment 3: The Methods section needs to be restructured to make it easily readable to a broader audience. Authors can use subheadings for data source, sampling design, target population, inclusion and exclusion criteria, variables, detailed descriptions of the design including randomization, blinding and masking, statistical analysis, etc. Adding a diagram showing the data flow would be helpful for readers.

Comment 4: The author already mentioned in the Statistical analysis section (Page 11; lines 27-44) that "Chi-square tests were used to test for differences in the proportion of messages delivered across the intervention and control groups, and Fisher's exact test was applied when the numbers in question were tiny." This analysis was insufficient to accurately measure the impact of the intervention. Sample description was not provided to assess whether the control and intervention groups were comparable in terms of their sociodemographic characteristics. Without this information, it is difficult to assess whether the intervention effect is due to the intervention or confounded. The authors used only some descriptive statistics and statistical tests. In a randomized trial, the effect size should be measured appropriately. I would recommend using a simple model that yields RR and robust CI, even with small counts. Such as Poisson regression with robust SEs, outcome and intervention vs. control, reporting RR, 95% CI, and p-value.

VERSION 1 - AUTHOR RESPONSE

Reviewer: Dr. Wilson Tumuhimbise, Mbarara University of Science and Technology

Thank you for your clear expert feedback Dr. Tumuhimbise.

- **Page 9, lines 31-34, was that 14th November same year 2023, what was the reason for that short time frame, was this a design issue and could this have had a significant effect on the study since participants were told that they wouldn't receive the airtime?**

Yes, it was the same year. We agreed with the government partners to run a short, limited time intervention targeting a very large sample of users to test our research hypothesis. The time limitation was to impose a limit on the payments in case the system was not effective in limiting screening by those who were not invited, thus enabling invited individuals to forward the message to low-risk non-targeted patients to earn airtime.

- **Page 13, line 3 and 5, define the term unique users**

Please note that unique users refers to users with a unique cellphone number. We use this term because in some cases, users may have attempted to complete the screening again in an attempt to get more airtime rewards. These observations were excluded from the

analysis. We had defined the term, but it was in a footnote. We have now brought this into the main text.

- **The fifth bullet under the strengths and limitations on Table 1, Is true that this entire study only 27 people in the intervention arm and 11 people in the Control arm began and completed the screening using the mobile phone out of the 1249 devices that acknowledged receipt?**

Yes, screening numbers were low, as can be seen in Table 1. In the control(treatment) group, 616(633) devices acknowledge receipt of the message, of which 8(20) started a screening and 3(7) completed it. We hope this is clearer now.

- **Was the unsolicited by design or coincidental? Given that people will always participate in studies they understand could this have contributed to this small number otherwise the effect of a good intervention like this may be deemed not effective because of the researchers' bias.**

Thank you for the question. The invitations were sent to people who already had a relationship with the Western Cape Department of Health and Wellness. We have noted this in the Methods section and again in the revised Discussion section. Even so, it is expected that unsolicited messages would yield a lower response rate. However, the question of response to such messages is important for many preventative or health promotion campaigns where mHealth interactions would be unsolicited but targeted at large sub-populations – similar to how we approached our sample in this case. We made this point clearer in the revised introduction. It would indeed likely be much easier to get substantial take-up among people who have already agreed to participate in a study. However, such take-up rates would be less likely to be achieved in a real-world setting, leading to potential issues with scalability. We believe it is important to understand whether an intervention such as this one is effective within the context of a real-world public healthcare system where patients may be difficult to contact, rather than in the ideal conditions of a conventional study environment. This is likely to be a much closer reflection of take-up in a real-world setting and whether an intervention would be effective at scale.

Reviewer: Dr. Emine Afşin, Bolu Abant İzzet Baysal University

- **Although the aim was to detect TB early, the number of patients who completed the screening was very low, and the limitations mentioned indicate that this study needs to be further developed**

Thank you for your feedback Dr. Afşin. Yes, this is a good summary and is reflected in our conclusions. We believe that it is important to document the finding that take-up of the screening was very low even with a small incentive. This may inform studies considering similar strategies to promote TB self-screening.

Reviewer: Dr. Mohammed Moinuddin, University of Lancashire
Results from a randomized controlled trial to evaluate whether airtime rewards through

unsolicited text messages increase the uptake of the TB Check self-screening platform. This is an interesting research topic; however, there are several caveats regarding the methodology and statistical analysis that require further attention. My specific observations are below.

Thank you Dr. Moinuddin for the clear and constructive advice.

1. **Sample size calculation and sampling - The authors mentioned "a power of 80%, a significance level of 5%, assuming a screening completion rate of 2% in the control group and 6% in the intervention group." Please clarify how you chose the 2% and 6% baseline rates. Please provide the reference for this statement. Please briefly explain.**

   Please let us clarify. These power calculations were not informed by literature given the dearth of literature on the topic and specific context, but informed by our own pilot to prepare the experiment. On March 15 2023 and before the RCT, 1000 test invitations (without incentives) were sent out. This was to test delivery success and screening uptake. No incentive was tested and these data only informed the power calculations. Note that out of these 1000 test invites, 46% were successfully delivered and 2% (out of 463 deliveries) led to the initiation of a screening, respectively. For the RCT experiment we assumed a MDE of 4pp induced by incentives as this was deemed the minimal, policy-relevant effect size by our team of researchers and stakeholders. We hope this makes our thinking more transparent. We have added details to the power calculations paragraph in the revised paper.

2. **The authors again mentioned in the footnote that the sample size was calculated for three groups with a success rate of 46%, which gives a total of 2452. However, the original description states that it was calculated based on a 2% success rate, with the number showing the same. I wonder how these two descriptions make sense. Please clarify.**

   Please compare above answer. We did factor in delivery rates to determine the required sample size. The power calculations yielded a required sample size (those who actually received the invitations) of 376 per group. This group size was divided by the SMS delivery success rate of 46% to obtain the required number of invites sent. The 46% was the SMS delivery success rate, while 2% was the initiated screening rate.

3. **Non-response in the form of non-delivery of text messages in this study was a major issue. Unfortunately, the authors did not consider this in their sample size calculation. Please justify.**

Please compare our answers above. We did factor in delivery rates based on our pilot.

4. **It is surprising how the randomization allocated the same number of patients in the two groups. This usually does not happen in simple randomization unless some mechanisms, such as block randomization, are adopted. In addition, please describe the participant selection procedure in detail.**
Apologies, details were missing in the paper. We used computer-based, simple and parallel randomization with equal allocation. Specifically, after the selection of 2500 cell phone numbers from the patient database (see paper for selection details). Phone numbers were individually randomized into two groups with equal group size. Essentially, every phone number was allocated a random number. The list of phone numbers was re-ordered based on the random number; the first(second) 1250 numbers were allocated to group 1(2). Thus forcing equal group size explains the same number of participants in both groups. We have noted this more clearly in the revised abstract and text.

5. **I wonder whether all the participants had sufficient language capability to understand the text messages. Please describe and compare the language by use and literacy rates by group in the study region. This may have been a strong confounder in this study.**

   The message was sent in the local language and recorded language preference of patients (as mentioned in the methods section). Because the team recognised that it was crucial to ensure that the message was simple and clear, we invested significant effort in this, including sharing the Xhosa, English and Afrikaans message with native language speakers for review. The literacy rate in Cape Town (the largest city in the Western Cape) is 93%, according to City of Cape Town figures. As of 2022, Black Africans had the highest illiteracy rates in South Africa, at 11.6%, while illiteracy rates were lowest among Whites at 0.4% (please note that these are the population group categories used by Statistics South Africa). These literacy rates are quite high, but we have added that literacy may have posed a barrier to take-up as a limitation.
   See:
   https://resource.capetown.gov.za/documentcentre/Documents/City%20research%20reports%20and%20review/SOCT_Report_Summary_2022.pdf#:~:text=Illiteracy%20levels%20The%20literacy%20level%20in%20Cape,to%20the%20global%20average%20of%2086%2C48%25%20(2019). [p. 18]
   https://lmi-research.org.za/wp-content/uploads/2024/07/DHET-FACTSHEET-7-7-3b-Adult-Literacy-2024-WEB.pdf [p. 4]

6. **The Methods section needs to be restructured to make it easily readable to a broader audience. Authors can use subheadings for data source, sampling design, target population, inclusion and exclusion criteria, variables, detailed descriptions of the design including randomization, blinding and masking, statistical analysis, etc. Adding a diagram showing the data flow would be helpful for readers.**

   Thank you, we restructured the Methods section and we have added the diagram (see Figure 1).

7. **The author already mentioned in the Statistical analysis section (Page 11; lines 27-44) that "Chi-square tests were used to test for differences in the proportion of messages delivered across the intervention and control groups, and Fisher's exact test was applied when the numbers in question were tiny." This analysis was insufficient to accurately measure the impact of the intervention. Sample description was not provided to assess whether the control and intervention groups were comparable in terms of their sociodemographic characteristics. Without this information, it is difficult to assess whether the intervention effect is due to the intervention or confounded. The authors used only some descriptive statistics and statistical tests. In a randomized trial, the effect size should be measured appropriately. I would recommend using a simple model that yields RR and robust CI, even with small counts. Such as Poisson regression with robust SEs, outcome and intervention vs. control, reporting RR, 95% CI, and p-value.**

Thank you for highlighting the concerns regarding the comparability of the intervention and control groups. We agree that this is important. Unfortunately, the large routine data we used for this work contained very few covariates, and does not enable us to effectively control for sociodemographic characteristics. In response to this suggestion, we did discuss the possibility of the Western Cape provincial Department of Health and Wellness team performing an additional analysis to check balance in terms of sociodemographic characteristics. However, it became apparent that this would be subject to a lengthy approval process in terms of patient data protection regulations. If the reviewer feels very strongly about this we are happy to go through this process. We have identified this as a limitation.

We also agree that the question you raise on the use of the Poisson regression is important to consider. Our data is binary so some readers may expect us to use the modified Poisson with robust SE. However, because our pilot study showed low response rates we opted for the use of simple and transparent statistical techniques such as the chi-square test, Fischer's exact tests, and unconditional exact tests such as Boschloo and Barnard that are robust to a low yield of positive responses [1-3]. These tests provide exact p-values without relying on large-sample approximations, making it ideal for instances such as ours where there were relatively few positive cases (8 vs 20 for screening initiation; 3 vs 7 for completion). In such

scenarios likelihood-based estimates such as the Poisson regression can frequently diverge from Fisher's exact tests because the Poisson estimation is reliant on asymptotics.

You also highlight that the recently updated CONSORT 2025 guidelines recommend reporting both absolute and relative effect sizes for binary outcomes (item 26). To fully align with CONSORT 2025, we have now added relative risk estimates with 95% confidence intervals to Table 1:

- **Device delivery**: RR = 1.03 (95% CI: 0.95-1.11, p=0.497)
- **Screening begun**: RR = 2.50 (95% CI: 1.09-5.73, p=0.035)
- **Screening completed**: RR = 2.33 (95% CI: 0.60-9.09, p=0.342)

We give greater prominence to the absolute measures, because there is a substantial methodological literature documenting that relative effect measures are frequently misinterpreted by clinicians, policymakers, and the public, often leading to overestimation of intervention effects [4-6]. Absolute effect measures (risk differences) are often more meaningful for decision-making, particularly in public health contexts [7,8].

References

1. Agresti A. 2013. Categorical Data Analysis. 3rd ed. Hoboken, NJ: John Wiley & Sons.
2. Campbell, I. 2007. Chi-squared and Fisher–Irwin tests of two-by-two tables with small sample recommendations. Statistics in Medicine, 26(19), 3661–3675. https://doi.org/10.1002/sim.2832
3. Lydersen, S., Fagerland, M. W., & Laake, P. 2009. Recommended tests for association in 2 × 2 tables. Statistics in Medicine, 28:1159–1175
4. Nuovo J, Melnikow J, Chang D. 2002. Reporting number needed to treat and absolute risk reduction in randomized controlled trials. JAMA, 287(21):2813-2814.
5. Schwartz LM, Woloshin S, Dvorin EL. 2006. Ratio measures in leading medical journals: structured review of accessibility of underlying absolute risks. BMJ, 333(7581):1248.
6. Gigerenzer G, Gaissmaier W, Kurz-Milcke E, Schwartz LM, Woloshin S. 2007. Helping doctors and patients make sense of health statistics. Psychol Sci Public Interest. 8(2):53-96.
7. Sedrakyan A, Shao J. 2012. Empirical assessment of reporting of absolute and relative measures in randomized trials: findings from systematic review protocol. Trials, 13:89.
8. Hopewell S, Chan A-W, Collins GS, Hróbjartsson A, Moher D, Schulz KF, Tunn R, Aggarwal R, Berkwits M, Berlin JA, Bhandari N, Butcher NJ, Campbell MK, Chidebe RCW, Elbourne D, Farmer A, Fergusson DA, Golub RM, Goodman SN, Hoffmann TC, Ioannidis JPA, Kahan BC, Knowles RL, Lamb SE, Lewis S, Loder E, Offringa M, Ravaud P, Richards DP, Rockhold FW, Schriger DL, Siegfried NL, Staniszewska S, Taylor RS, Thabane L, Torgerson D, Vohra S, White IR, Boutron I. 2025. CONSORT 2025 statement: updated guideline for reporting randomised trials. BMJ. 389:e081123. doi:10.1136/bmj-2024-081123
